# African Primary Care Research: Qualitative interviewing in primary care

**DOI:** 10.4102/phcfm.v6i1.632

**Published:** 2014-06-05

**Authors:** Steve Reid, Bob Mash

**Affiliations:** 1 Primary Health Care Directorate, University of Cape Town, South Africa; 2 Family Medicine and Primary Care, Stellenbosch University, South Africa

## Abstract

This article is part of a series on African Primary Care Research and focuses on the topic of qualitative interviewing in primary care. In particular it looks at issues of study design, sample size, sampling and interviewing in relation to individual and focus group interviews. There is a particular focus on helping postgraduate students at a Masters level to write their research proposals.

## Abstrait

**Recherche africaine dans le domaine des soins primaires: interviews qualitatives dans le domaine des soins primaires**. Cet article fait partie d'une série sur la Recherche africaine dans le Domaine des Soins primaires et se concentre sur le thème des interviews qualitatives dans le domaine des soins primaires. On y examine la conception de l’étude, la taille des échantillons, les échantillons et les interviews des individus et groupes cibles. On s'efforce particulièrement à aider les étudiants licenciés au niveau de la Maitrise à rédiger leurs propositions de recherche.

## Introduction

This article is part of a series on Primary Care Research and deals with qualitative interviewing and the issues to consider when writing your research proposal. Other articles in the series outline how to write a proposal, ethical considerations, how to perform a literature review and qualitative data analysis, amongst other topics.

Qualitative research is helpful with regard to exploring a phenomenon or a behaviour that is poorly understood, in order to interpret it from the perspective of those who have a direct involvement.^1^ In contrast to quantitative studies, in which certain variables are controlled and others compared, qualitative studies create deeper understanding by allowing for contextual issues to be included in the data, even including the background and person of the researcher and the process of data collection itself.

Mixed-methods research, in which both quantitative and qualitative methods are used, combines the strengths of both approaches by describing the whole picture as fully as possible.^2^ The sequence is debatable: exploratory qualitative studies can provide insight into a phenomenon and the important variables in that context that can then be quantified in a subsequent study. Qualitative process evaluation can also provide insight into why a particular intervention, that might have been observed originally or measured quantitatively, works or does not work in that context. The two types of studies produce different data: quantitative studies produce numerical data and statistics that are illustrated in tables and graphs, whereas qualitative studies produce textual data and themes that can be illustrated by selected quotes and conceptual diagrams.

## Study design

Most qualitative studies are descriptive in nature and use interviews, participant observation or ethnography (the study of a phenomenon or disease in a particular cultural framework, usually over a period of time) to gain an in-depth understanding of behaviour.^3, 4^

The two interview methods used in qualitative research are individual interviews of key informants and focus group discussions (FGDs). The same principles of the qualitative approach apply to both forms of interview, which aim to explore the interviewee's perspective of the world and the meaning that they make of it.^5^

FGDs are a form of group interview that capitalise on communication between research participants in order to generate data on collective views, as well as the meanings that lie behind those views. They are used widely to examine people's experiences of disease and of health services and are an effective technique for exploring the attitudes or needs of staff. Although group interviews are often used simply as a quick and convenient way to collect data from several people simultaneously, focus groups explicitly use group interaction as part of the method. This means that instead of the researcher asking each person to respond to a question in turn, people are encouraged to talk to one another: asking questions, exchanging anecdotes and commenting on each other's experiences and points of view. The method is particularly useful for exploring people's knowledge and experiences and can be used to examine not only *what* people think, but *how* they think and *why* they think that way. They also have advantages for researchers in the field of health and medicine since they do not discriminate against people who cannot read or write and they can encourage participation from people who are reluctant to be interviewed on their own or who feel they have nothing to say.

Another aspect of study design is the degree of structure one chooses, from structured to semi-structured, to unstructured or open-ended interviews. Structured interviews are, essentially, verbally-administered questionnaires in which a list of predetermined questions are asked, with little or no variation and with no scope for follow-up questions to responses that warrant further elaboration.

Most qualitative researchers design semi-structured interviews using a predetermined interview guide that sets out the broad issues that are assumed to be important through a sequence of open-ended questions. Developing an interview guide requires careful consideration of the main research question and its constituent parts; and particular attention needs to be given to their phrasing. The initial open question in the interview guide can reflect the overall research question of the study almost directly, whilst subsequent questions will probe specific aspects of the issue under discussion. Questions should move from general to more specific questions and the order should be relative to the importance of issues in the research agenda. It is important that each question is structured in an open-ended way so as to avoid one-word answers and encourage elaboration.

Unstructured or open-ended interviews do not reflect any preconceived theories or ideas and require greater skill. Such an interview may simply start with an opening question such as ‘*Can you tell me about your experience of x?*’ and will then progress, based primarily upon the initial response, using techniques described below under the heading ‘The Interview’.

## Sample size

The number of interviewees does not need to be as large as in quantitative studies. The idea is to try to get an in-depth understanding of a phenomenon from a few perspectives, each of which is unique, as opposed to determining the average and the range for a predetermined set of variables. A sample size of one has even been used, since one person's particular experience can raise a wide range of issues that may not have been considered previously.

Generally for individual interviews, a sample size of between five and 15 interviewees is adequate to obtain a sufficient range of responses and experiences, until a level of so-called data ‘saturation’ is reached, beyond which no significantly new information is being produced. This can be discussed by the research team after each batch of five interviews has been completed and a decision can be made regarding whether or not to continue. An alternative approach to the size of the sample is to perform a preliminary analysis of the data after each interview, developing new ideas and hypotheses in an iterative process which can then be tested in subsequent interviews.

For FGDs, a group size of between five and eight people is advised, but they can work successfully with as few as three and as many as 15 participants. It may, however, be necessary to invite more than eight in case some do not come. A diversity of views is useful, but it is important to be aware of power dynamics in a group, such as between nurses and doctors. With gender-sensitive issues, for example, it may be better to hold FGDs with women and men separately. The profile and diversity of interviewees needs to be carefully considered, as it is important to recruit key informants who have the position and experience that will enable you to gain a perspective of a whole system.

When writing your research proposal it is usual to state at least the initial number of interviews you plan to perform and the rationale for this, as well as how you will determine if you have interviewed sufficient people in order to explore the phenomenon and meet your objectives.

## Sampling

Representivity is not the issue in qualitative research, so random sampling is unnecessary. Convenience sampling can be used, as determined by the context of the recruitment process, but a more directed approach gives better results. Purposive sampling allows you to choose to interview those who are key informants by virtue of their position, or who are known for their opinions and views and are not afraid to voice them. These are so-called ‘information-rich’ participants, as compared with those who are known to be quiet or withdrawn. Examples of purposive sampling include:^6^

- *Extreme case sampling:* A controversial figure in a particular group or community, for example, is useful in drawing out divergent views on an issue and should be specifically sought out for an FGD or interview. Learning from unusual manifestations of the phenomenon can be helpful, for example, when selecting outstanding successes or notable failures to interview.
- *Criterion sampling:* Inclusion and exclusion criteria can be drawn up according to the purposes of the study and purposive sampling allows one to choose participants who will potentially be the most informative.
- *Snowball sampling:* A ‘snow-balling’ technique can be used to find further participants through those recruited initially, asking particularly for those known to have strong views on the topic under study. It is also possible to ‘sample as you go’, purposively choosing further subjects as you learn more about the issues.
- *Purposeful random sampling:* This is useful when the potential number of people who could be interviewed is too many to cope with practically and all are of equal interest. Remember, though, that in this case random sampling is being used for a different purpose than in quantitative study designs. The aim is not to produce data that is representative of a larger population, but rather to understand a few instances that help us to appreciate the variety and limits of a phenomenon.

It is important that the process of recruitment of interviewees is entirely voluntary and without coercion, as this may affect the quality of the information collected.

In considering who could be interviewed for a particular research question, a number of criteria should be borne in mind:

- The total number of interviewees.
- The profile and diversity of the interviewees.
- The likelihood of obtaining useful information on the phenomenon in which you are interested.

When writing your research proposal you should describe your sampling strategy and the specific criteria used to select people in purposive sampling. The practical way in which these criteria will be applied in the field should also be described.

## Data collection

### The interviewer

Qualitative interviewing requires specific skills which can be developed and learnt, but not everyone makes a good interviewer. Do not make the assumption that it is an easy task, or that the researcher should necessarily be the interviewer. Specific training in qualitative interviewing may be required by members of the research team who plan to do the interviewing themselves; this should be arranged in good time. At the very least, the interview guide and process of interviewing should be piloted or role-played by the interviewers. Alternatively, it may be preferable to engage the services of a professional interviewer who has no vested interest in the research topic, particularly if there is a language barrier between the researcher and the interviewees.

The person who is the researcher or interviewer is important in qualitative research and should be ‘visible’ in the report. Participants asked the same question will give different responses to different people depending on who they are, or who they perceive them to be. So the actual results of a qualitative study are influenced directly by the person of the researcher, not only their level of skills but also their background and pre-existing views. Every interviewer, however skilled or professional, brings a certain bias with regard to the topic under discussion into an interview, which is a recognised feature of qualitative research. One method for minimising this bias is for the principal investigator or academic supervisor to interview the interviewer(s) on what they expect to find in response to the research question and to record this interview. This then forms part of the dataset and is used in the analysis, along with the interviews of the research participants. The effect of this process is to make explicit the existing ideas, position and bias of the interviewer(s) with respect to the topic under discussion, so that they are more consciously aware of the moments in the interviews when they have strong feelings or opinions, positive or negative, in response to what the participants are saying.

The facilitation of FGDs requires a greater level of skill, as they are more complex than individual interviews and, again, specific training and practice are helpful. It is often necessary to hold several opposing or conflicting views in the group in tension during the discussion and to create a space for those who feel less confident to express themselves clearly. When there are divergent views or opinions within a group, it is important to acknowledge these clearly when summarising, without assuming that there should be consensus or agreement. These are high order skills that require equanimity or balance, particularly when the discussion becomes heated or emotional, whilst at the same time keeping in mind the overall direction of the discussion. When to intervene with a participant who is dominating the discussion, for example, or how to encourage those who are reticent with regard to expressing their views, are skills that can only be acquired through practice and reflection. Reviewing the transcripts or the actual videos of FGDs is a powerful method of reflection and learning, which is best done through non-judgemental feedback by peers or an experienced interviewer. As with any skill, the more often one practises it, the more comfortable one becomes with the process and the better the quality of information that can be collected which truly reflects the participants’ views.

### Inviting the participants

Participants should be invited well in advance and given the clear option of declining the invitation without prejudice. It is important to state up front that refusing to participate in the interview will not influence the way that they are dealt with, for example, as patients or members of staff who may be reticent to speak freely about issues that could potentially affect them personally. Each potential participant should be sent an information sheet about the study, as well as the consent form, in advance, including the standard details of confidentiality and protection of privacy. It is important to note, however, that FGDs cannot guarantee anonymity, because the other members of the FGD cannot be bound by confidentiality agreements. If it is appropriate, transport costs for attending the interview can be reimbursed at a standard rate, but no other incentives are usually offered, apart from tea or refreshments when they arrive.

### Setting up the interviews

It is useful to devote some attention to setting up the venue for the interviews optimally, as this may have a direct effect on the quality of the data collected. A venue that is neutral is preferable, separate from the environment of the issues under discussion, so that it does not set up unnatural power dynamics (e.g. a board room that intimidates participants) or provoke potentially uncomfortable feelings or reactions. Participants need to be invited well in advance so that they are able to clear their schedules for the interview. In a work environment, it is often good to use a lunch hour or an after-hours timeslot, unless it has been clearly negotiated and approved by their managers, so that participants are not anxious about getting back to work. If you are interviewing patients, make sure that they are not inconvenienced in terms of missing their appointments or losing their place in a queue.

The logistical arrangements are crucial, including the seating arrangements in the venue, the tea or refreshments and the setting up of audio- or video-recording apparatus. With FGDs, a circle of chairs in a flat venue without desks or tables is optimal. For individual interviews, it is better to avoid talking across a desk, rather sitting alongside the interviewee. It is preferable not to take notes as this can distract the interviewees’ attention, apart from very brief reminders for effective summary. Ask participants to switch off cellphones and try to minimise other possible interruptions.

It is worth investing in good recording equipment and to check it properly before and during the interview. There is nothing worse than finding that the interview or FGD which took an enormous amount of effort to set up, failed to be recorded adequately for technical reasons, with the result that the data is lost.

### The interviews

After welcoming the interviewees, start by explaining the procedure and the length of time that it is expected that the interview will take, as well as the purpose of the study. You will need 60 minutes for a focus group and between 30 and 60 minutes for an individual interview. Give enough information about the study, repeating what is written in the information sheet, without actually asking the research question before the discussion begins, and allow for questions of clarification. Once participants are satisfied and prepared to continue, ask them to sign the consent form. At the same time, it is useful for each participant to complete a basic demographic identification form, without names, so that the profile of the participants can be described when writing up the study.

Explain the role of an observer or camera operator, if there is someone helping with the recording, and request that cellphones be put on silent mode.

Begin the interview or focus group by requesting everyone in the room to contribute to open discussion without restriction in response to the questions, including examples or personal experiences. As some participants will not be accustomed to open-ended interviews, they will need to be encouraged to voice their opinions spontaneously rather than waiting to respond to a specific question.

Start with the main overall research question that is phrased in as open a way as possible, so as to allow the participants to understand the ultimate aim of the interview before going into detail. Wait for the initial responses without interrupting. Facilitate the discussion by summarising these initial responses in your own words, which gives you an opportunity to restate the research question. Don't be in a rush to go on to the next question in the interview guide, but rather wait for the participants to produce their own priorities in their own words, using encouraging body language. Ask for examples to illustrate what they have been saying, or ask a question such as: ‘*Can you tell us more about that?*’ or ‘*Can you give us an example from your own experience?*’. Don't be afraid of short silences, as people are often just thinking about what has been said or catching up in their heads.

The two main tools of facilitation are clarifying and summarising. Clarification seeks to expand a notion by giving it different words and descriptions, as in: ‘*If I understand you correctly, you mean* …*’* or ‘*Can you explain what you mean by* …?’. It also includes requesting examples or experiences to elaborate on a point. Asking ‘why’ can stimulate people to think about an issue at a deeper level, for example, ‘*When you said you felt x, why was that?*’.

Summarising means bringing together a series of inputs and reflecting them back to the participants, using their own words as far as possible: ‘*So to summarise what we have heard so far, it seems that* … ’. This is also an opportunity in a FGD to reflect divergent views, as in: ‘*It appears that there are two different perspectives on this issue in the group:* … ’. Summarising is also a technique of reflective listening. The facilitator should summarise the discussion at several points in the course of the interview, trying to capture as accurately as possible the main issues that have been put forward. The first summary reflection should be given within 10 minutes of the start of the interview and a final summary reviewing all the issues should be given toward the end. An accurate summary by the facilitator reassures the participants that what they have said has been heard, received and understood. If it is not accurate, they then have an opportunity to correct it, or they can add to it or embellish it with an example. Reflecting back an accurate summary releases the participants to think about what has not been said so far and to offer further information or a whole new idea. In other words, summaries should not close off the discussion, but rather invite more input, so they should be phrased in a tentative way rather than in a definitive manner that cannot be challenged.

An interview guide can be both useful and a hindrance. In inexperienced hands it can be turned into a questionnaire that becomes a barrier to free discussion between the interviewer and participants and may shape the discussion toward the researcher's bias. It should rather be seen as a supportive tool, for reference if the discussion needs some stimulation, but only after the techniques of clarifying and summarising have been used to elaborate on the main research question. Above all, it should only contain open-ended questions that help to remind the interviewer of important components of the research question that are likely to be raised. But it is better if the main issues are brought up spontaneously by the participants through active facilitation. If they do not arise spontaneously, it could be deduced that the participants did not regard them as important enough to mention.

Stay with the ideas and experiences of the interviewees until they have been understood and explored even if they are not what you expected, as long as it is within the focus of the study. Pay particular attention to viewpoints that contradict your own views or others in the group, as there is a tendency to not hear these. Similarly, when interviewees bring up issues that you feel strongly about yourself, be aware of your own responses that may bias the discussion in one particular direction. Never correct an interviewee during an interview, or offer advice. Also, avoid answering direct questions from the interviewees about the content. Rather reflect these back to them by asking: ‘*What is your opinion on that issue?*’.

In an FGD, it is often necessary to spread the questions around the group, or to use body language to turn to members of the group who have been silent up to that point, anticipating their input with a reflection such as: ‘*What do the rest of you think of this issue?*’. Invite specific participants’ contributions by name or, after a strong contribution by one participant, ask the question: ‘*Does everyone else feel that way, or are there others who feel differently?*’. Look for issues in which there is a divergence of opinion in order to stimulate more discussion, as in: ‘*So Mr X says this and Ms Y maintains that: what is the actual situation?*’.

The final summary by the facilitator, if well done, may not be the actual end of the interview, because participants may be reminded of other points that have not been raised at all. So be prepared to be flexible, but at the same time stick to the agreed time for the interview. When the allotted time is up, thank all the participants for their participation and give them some idea of what the next steps are. If a feedback session is planned, then that is an opportunity to arrange it.

Conclude interviews with care and respect for the issues and emotions that may have been raised. Any unresolved emotional issues need to be dealt with through appropriate referral or another suitable process. It is often necessary to clarify how the information will be analysed and used, with a commitment to feeding back the reports or papers that arise from the study.

In your research proposal it is important to describe your interview guide and often to include it in full as an appendix. The practical details of who will conduct the interview, how language issues will be dealt with, how we can be sure that they will be a skillful interviewer, how they will be reflexive about their own prior perspective, how people will be invited, where the interviews will be held, how they will be recorded and any other practical arrangements (see Box 1) should be described in the data collection section of your research proposal.

BOX 1 Practical checklist for conducting qualitative interviews.

1. Use purposive sampling to choose information-rich participants.
2. Invite the participants well in advance.
3. Send them the information sheet and consent form.
4. Offer to reimburse transport costs if appropriate.
5. Set aside adequate time for the interview.
6. Choose and secure a neutral venue where there will be no interruptions.
7. Set up the audio- or video-recording apparatus and test it.
8. Offer tea or refreshments when the participant(s) arrive(s).
9. Request their full attention without interruptions (e.g. cellphones).
10. Explain the purpose of the study and the process of the interview.
11. Obtain their signed consent including for recording.
12. Request participants to complete an anonymous demographics sheet.
13. If there is an observer, clarify their role with the group.
14. Start with the main research question.
15. Develop and use an interview guide of open-ended questions.
16. Clarify responses and issues as they arise.
17. In an FGD, spread the questions around the group.
18. Summarise frequently, as well as toward the end of the time.
19. Conclude the interview or FGD with care and respect for the participants.
20. Refer participants with any unresolved issues appropriately, if necessary.

FGD, focus group discussion.

## Open-ended interviewing in primary care

There is much to learn from qualitative research that has relevance to clinical practice in primary care.^7^ In contrast to the closed questions of traditional history-taking, as is taught to medical students, the open-ended interview style used in qualitative research resonates with the more relational approach in family medicine and primary care.

Open-ended interviewing skills as practised in qualitative research can add a rigour to the consultation process in primary care by teaching practitioners who undertake interviews how to listen actively to what the patient is saying. The attitude of tuning in to the patient without interruption, giving one's full attention to exactly what is being said and meant, as well as what is not being said, can reveal the patient's real agenda within the first few minutes of an interaction.^8^ In contrast to expectations, active listening does not necessarily take more time and the qualitative interviewing techniques outlined above, namely, careful listening, clarifying and summarising, can actually lead to shorter and more efficient consultations, since the real issues of concern to the patient become apparent early on. Clarifying the patient's experience of the illness or problem by following their lead, avoids the interrogative style of standard history-taking that uses closed questions and puts the patient on the defensive. Summarising the patient's subjective experience of the illness in the practitioner's words can be extremely reassuring to those who feel distressed or alienated, as they finally feel that someone has listened and understood them. A skilled listener is far more efficient than a practitioner who asks all the correct routine questions, because they are able to understand the patient's agenda more quickly and can make a better assessment and plan.

## Conclusion

This article has outlined the key issues to consider when planning a qualitative study using individual or focus group interviews. The article should assist you with writing the methods section of your research proposal, particularly the study design, sample size, sampling and data collection section. Qualitative data analysis is covered in another article in this series on Primary Care Research.
